# Insights into how Spt5 functions in transcription elongation and repressing transcription coupled DNA repair

**DOI:** 10.1093/nar/gku333

**Published:** 2014-05-09

**Authors:** Wentao Li, Cristina Giles, Shisheng Li

**Affiliations:** 1 Department of Comparative Biomedical Sciences, School of Veterinary Medicine, Louisiana State University, Baton Rouge, LA, USA; 2 Department of Biological Sciences, Louisiana State University, Baton Rouge, LA, USA

## Abstract

Spt5, a transcription elongation factor, and Rpb4, a subunit of RNA polymerase II (RNAP II) that forms a subcomplex with Rpb7, play important roles in transcription elongation and repression of transcription coupled DNA repair (TCR) in eukaryotic cells. How Spt5 physically interacts with RNAP II, and if and/or how Spt5 and Rpb4/7 coordinate to achieve the distinctive functions have been enigmatic. By site-specific incorporation of the unnatural amino acid *p*-benzoyl-L-phenylalanine, a photoreactive cross-linker, we mapped interactions between Spt5 and RNAP II in *Saccharomyces cerevisiae*. Through its KOW4-5 domains, Spt5 extensively interacts with Rpb4/7. Spt5 also interacts with Rpb1 and Rpb2, two largest subunits of RNAP II, at the clamp, protrusion and wall domains. These interactions may lock the clamp to the closed conformation and enclose the DNA being transcribed in the central cleft of RNAP II. Deletion of Spt5 KOW4-5 domains decreases transcription elongation and derepresses TCR. Our findings suggest that Spt5 is a key coordinator for holding the RNAP II complex in a closed conformation that is highly competent for transcription elongation but repressive to TCR.

## INTRODUCTION

RNA polymerases (RNAPs), which carry out transcription in all living organisms, are highly conserved at the level of sequence, structure, function and molecular mechanisms (1). The most studied eukaryotic RNAP is RNAP II that consists of 12 subunits (Rpb1-12). Rpb4 and Rpb7 form a dissociable stalk structure, whereas the rest subunits form the core RNAP II (2). An RNAP interacts with different factors during transcription initiation and elongation (1). The binding sites for initiation and elongation factors on an RNAP may overlap and the binding of the factors to RNAP is mutually exclusive, which ensures an efficient swapping of factors and may assist RNAP during promoter escape (3).

NusG/Spt5 family proteins are universally conserved transcription elongation factors that play pivotal roles in transcription and transcription related processes by binding to RNAP and interacting with other transcription-associated factors (4). Bacterial NusG and archaeal Spt5 proteins contain an N-terminal NGN domain and a C-terminal KOW domain (Supplementary Figure S1) (5,6). Eukaryotic Spt5 proteins are much larger (1063 and 1087 residues in *Saccharomyces* *cerevisiae* and humans, respectively) and more complex, consisting of an N-terminal acidic domain, an NGN domain, multiple KOW domains and a C-terminal repeat (CTR) domain (4). The NGN domain of archaeal and eukaryotic Spt5 associates with Spt4, a relatively small zinc-binding protein (61, 102 and 117 residues in *Pyrococcus* *furiosus*, *S. cerevisiae* and humans, respectively), to form a heterodimeric Spt4/5 complex.

The archaeal Spt4/5 has been crystallized (7,8) and the structural model of archaeal RNAP-Spt4/5 complex has been reconstructed based on analyses of cryoelectron microscopy single particles (8). The archaeal Spt4/5 complexed with the clamp domain of archaeal RNAP has also been crystallized (9). In the archaeal RNAP clamp-Spt4/5 structure, the NGN domain of Spt5 directly interacts with the RNAP clamp, whereas Spt4 interacts with the other side of the NGN domain (9). Furthermore, the crystal structures of the NGN domain of *S. cerevisiae* and human Spt5 bound to Spt4 have been solved (10,11). However, it is still very challenging to solve the structures of complete eukaryotic Spt4/5, either alone or in complex with RNAP II, presumably because the fairly large eukaryotic Spt5 proteins contain multiple disordered or unstructured regions. Due to the lack of structural information, how Spt5 functions in transcription elongation and transcription related processes in eukaryotic cells has been enigmatic.

Nucleotide excision repair (NER) is a DNA repair pathway that removes a wide variety of bulky and/or helix-distorting lesions that generally obstruct transcription, such as UV-induced cyclobutane pyrimidine dimers (CPDs) (12,13). Transcription coupled repair (TCR) is an NER subpathway dedicated to rapid removal of DNA lesions in the transcribed strand (TS) of actively transcribed genes (14). TCR is believed to be initiated by an RNAP stalled at a lesion in the TS of a gene being transcribed. The TCR mechanism in bacteria has been elucidated in molecular details (15–19). Mfd and UvrD, two DNA helicases/translocases, have been shown to play important roles in TCR in *Escherichia coli*. Mfd binds to the β subunit of RNAP stalled at a lesion and displaces the complex by pushing it forward (15,17–19). Concurrently, Mfd recruits UvrA to the exposed lesion site to facilitate NER (19). On the other hand, UvrD binds RNAP during transcription elongation and forces RNAP to backtrack along DNA, thereby exposing DNA lesions for access of NER machinery (16). The biochemical mechanism of TCR in eukaryotic cells is still enigmatic. In the budding yeast *S. cerevisiae*, Rad26, a DNA-stimulated ATPase that is homologous to the human CSB protein, plays an important role in TCR (20). However, Rad26 is dispensable for TCR in cells lacking Rpb4 (21) or Spt4 (22). Rad26 is also partially dispensable for TCR in cells lacking the CTR domain of Spt5 (23) or any subunit of the 5-subunit RNAP II associated factor 1 complex (PAFc) (24). Therefore, TCR appears to be repressed by certain factors that are normally involved in transcription elongation and Rad26 facilitates TCR by antagonizing the repression (13). How these factors repress TCR and if and/or how they coordinate in the repression remain to be elucidated.

To gain insights into the mechanisms that underlie the functions of Spt5 in eukaryotic cells, we mapped site-specific interactions between Spt5 and RNAP II in *S. cerevisiae*. We found that Spt5 interacts with the clamp, protrusion, wall and Rpb4/7 stalk domains of RNAP II. The binding sites of Spt5 on RNAP II partially overlap with those of the transcription initiating factor TFIIE. Disruption of the interactions between Spt5 and Rpb4/7 by deleting the Spt5 domains that extensively interact with Rpb4/7 decreases transcription elongation and derepresses TCR. Our results suggest that Spt5 is a key coordinator for holding the RNAP II complex in a closed conformation that is highly competent for transcription elongation but repressive to TCR.

## MATERIALS AND METHODS

### Yeast plasmids and strains

Plasmid pLH157 bearing the genetically engineered *E. coli* tRNA_CUA_ and tyrosyl-tRNA synthetase genes (Supplementary Figure S2A) was obtained from Dr Steven Hahn. Multi-copy *LEU2* plasmids bearing genes of interest (GOI) (Rpb1, Rpb2, Rpb4, Rpb7 and Spt5) with a TAG codon replacing a desired amino acid codon were created using plasmid pESC-LEU (Stratagene) as vector (Supplementary Figure S2B). The *LEU2* gene on the original vector contains the leucine tRNA gene tRNA_3_^Leu^, which starts −463 nucleotides upstream of the start codon of the *LEU2* gene (25). We found that the tRNA_3_^Leu^ on the vector greatly compromises incorporation of *p*-benzoyl-L-phenylalanine (Bpa) into the protein of interest. The tRNA_3_^Leu^ gene was therefore inactivated by removing the sequence between the HpaI and SfoI sites in the gene. To increase detection sensitivity, the Flag tag contained in the original vector was converted to 3×Flag tag by inserting two Flag sequences between the SacI and BglII sites. The *GAL1–10* promoter sequence on the vector was removed and replaced with the GOI encompassing their native promoters and coding sequences with a TAG codon replacing an amino acid codon of interest. All mutations were confirmed by DNA sequencing.

Plasmids pRS416-RPB1, pRS416-RPB2, pRS416-RPB4, pRS416-RPB7 and pRS416-SPT5 were created by inserting the whole respective genes including the promoter, coding sequence and 3′ terminator sequences into the multiple cloning site of the single-copy centromeric *URA3* plasmid pRS416 (26). Plasmid pNAT-SPT5ΔCTR, which encodes 3×Myc-tagged CTR-deleted Spt5, was created by replacing the *LEU2* gene in plasmid pSPT5/CTRΔ (23) with the NAT (nourseothricin) gene. Plasmids pRS415-SPT5, pRS415-SPT5ΔKOW4 and pRS415-SPT5ΔKOW4-5, encoding the full-length, KOW4 deleted (deleting amino acids 706–765) and KOW4-5 deleted (deleting amino acids 706–848) Spt5, respectively, were created by inserting appropriate polymerase chain reaction (PCR) fragments of the *SPT5* gene into the EagI and BamHI sites of the single-copy centromeric *LEU2* plasmid pRS415 (26). To tag a genomic GOI with 3×Myc, plasmid p3MYC-KanMX was created by replacing the 3×Flag sequence in plasmid p3FLAG-KanMX (27) with a 3×Myc sequence.

All yeast strains used in this study are derivatives of BJ5465 (*MATa ura3-52 trp1 leu2Δ1 his3Δ200 pep4::HIS3 prb1Δ1.6R can1*). Deletion of genes was performed using procedures previously described (21). To delete the genomic *RPB1*, *RPB2*, *RPB4*, *RPB7* and *SPT5* genes, the cells were first transformed with plasmids pRS416-RPB1, pRS416-RPB2, pRS416-RPB4, pRS416-RPB7 and pRS416-SPT5, respectively. The 3×Myc tagging of a genomic gene was achieved by using PCR fragment amplified from plasmid p3MYC-KanMX. The deletion and tagging of a gene was confirmed by PCR.

The pGOI-TAG plasmids encoding Rpb1, Rpb2, Rpb4, Rpb7 and Spt5 with a TAG codon replacing a desired amino acid codon were transformed into respective yeast strains (Supplementary Table S1). Plasmids pRS415-SPT5, pRS415-SPT5ΔKOW4 and pRS415-SPT5ΔKOW4-5 were transformed into yeast strains whose genomic *SPT5* gene had been deleted and complemented with pRS416-SPT5. The transformed cells were selected with 5-fluoroorotic acid (5-FOA), which is toxic to cells with a functional *URA3* gene, to select for cells that had lost the *URA3* (pRS416) plasmids bearing the respective wild type GOI. The loss of the *URA3* plasmids and the gain of the *LEU2* plasmids in the respective transformed yeast cells were confirmed by PCR.

### Detection of cross-linking of Bpa-substituted proteins

Yeast cells having plasmid pLH157 and the pGOI-TAG plasmids encoding Bpa-substituted proteins of interest were grown at 30°C in synthetic dextrose (SD) medium containing 0.5 mM Bpa (Bachem) to late log phase (*A*_600_ ≈ 1.0) and harvested. The harvested cells from 15 ml of culture were washed twice with ice-cold H_2_O, resuspended in 20 ml ice-cold 2% glucose, and split into two aliquots. One aliquot was kept on ice and the other was transferred into a glass petri dish (10 cm in diameter) and irradiated with 365 nm UVA for 15 min (total dose of 54 000 J/m^2^) on ice. The UVA source was an array of 12 fluorescent black light tubes (15W, T8 and 22 inches in length, Utilitech) mounted on a home-made wooden structure (the light tubes were 5 cm apart on the structure). The UVA source gave a dose rate of 60 J/m^2^/s at a distance of 13 cm. The cells were harvested, resuspended in 400 μl of 20% trichloroacetic acid and broken by vortexing with 400 μl of glass beads for 30 min. The proteins were pelleted by centrifugation at 16 000 × g for 15 min at 4°C, washed with ice-cold 80% acetone, resolved on sodium dodecyl sulphate-polyacrylamide gel electrophoresis (SDS-PAGE) gels and subjected to Western blot. Specific SDS-PAGE conditions for Western blot detection of cross-linkings between different proteins are presented in Supplementary Table S2. Rpb1 on the blots was detected with 8WG16 (Neoclone), which recognizes the CTRs of Rpb1. 3×Flag and 3×Myc-tagged proteins were detected with anti-Flag M2 (Sigma) and anti-c-Myc (Genscript) antibodies, respectively. Blots were incubated with SuperSignal^®^ West Femto maximum-sensitivity substrate (Pierce) and scanned with the VersaDoc Imaging System (BioRad).

### Detection of co-immunoprecipitation of Spt5 and Rpb4 with core RNAP II and cellular levels of Rad26

For detection of co-immunoprecipitation of Spt5 and Rpb4 with core RNAP II, 90 ml of log phase yeast cells were harvested and resuspended in 0.6 ml of chromatin preparation buffer (50 mM HEPES pH7.8, 150 mM NaCl, 0.5% NP-40, 0.25% Triton X-100, 10% glycerol and protease inhibitors). The cells were broken by vortexing with acid-washed glass beads, and the chromatin fractions were collected by centrifugation at 20 000 × *g* for 10 min at 4°C. The chromatin pellet was solubilized in 250 μl of immunoprecipitation buffer (50 mM HEPES pH 7.5, 140 mM NaCl, 1% Triton X-100, 0.1% sodium deoxycholate, 1 mM EDTA, 10 mM NaF, 10 mM Na_4_P_2_O_7_ and protease inhibitors) by sonication with a Bioruptor (Diagenode) for 15 min (30 s on and 30 s off). The sample was clarified by centrifugation and the supernatant was transferred to a fresh tube. The sample was added with 1 ml of immunoprecipitation buffer and SDS to a final concentration of 0.1%. Fifty microliters of the sample was saved as ‘input’ and the remaining was mock-immunoprecipitated (without addition of antibody) or immunoprecipitated with 4 μg of 8WG16. The levels of Rpb1, Rpb4 and 3×Myc-tagged Spt5 were detected with 8WG16, 2Y14 (Neoclone) and anti-Myc antibodies, respectively, on western blots.

For detection of the cellular levels of Rad26, log phase yeast cells were harvested and the whole cell extracts were prepared using the glass beads and trichloroacetic acid method (see above). The levels of 3×Flag-tagged Rad26 and Rpb1 were detected using anti-Flag and 8WG16 antibodies, respectively, on a western blot.

### Tests of temperature, UVC and mycophenolic acid (MPA) sensitivities

Yeast cells were grown at 30°C in SD medium to saturation, and sequential 10-fold serial dilutions were made. For temperature sensitivity test, the diluted samples were spotted onto YPD (1% yeast extract, 2% peptone and 2% dextrose) plates and incubated at 25, 30 and 37°C. For UVC sensitivity assay, the diluted samples were spotted onto YPD plates, irradiated with different doses of 254 nm UV light (from a 15W UV germicidal bulb, General Electric) and incubated at 30°C in the dark. For mycophenolate sensitivity assay, the diluted samples were spotted onto SD plates containing different concentrations of MPA and incubated at 30°C. After 3–8 days of incubation the plates were photographed.

### Chromatin immunoprecipitation (ChIP) assay

ChIP assays were performed as described previously (28). Briefly, yeast cells were grown in SD medium to late log phase (*A*_600_ ≈ 1.0), cross-linked with 1% formaldehyde and lysed by vortexing with glass beads. The cell lysates were sonicated by using a Bioruptor (Diagenode) to shear the chromatin DNA to an average size of 200 bp and clarified by centrifugation at 4°C. An aliquot from each of the clarified lysates was saved as an input. The remaining lysates were immunoprecipitated with anti-Rpb1 antibody 8WG16 or mock-immunoprecipitated. DNA fragments corresponding to different regions of the *RPB2* gene in the input, immunoprecipitated and mock-immunoprecipitated samples were quantified in triplicates by using real-time PCR. Primers used for amplifying the different regions of the *RPB2* gene are shown in Supplementary Table S3. The number of molecules in each immunoprecipitated sample was subtracted by that in the corresponding mock-immunoprecipitated sample (generally ∼5% of the immunoprecipitated sample) and then normalized to that in the corresponding input. Each ChIP assay was repeated three times. The levels of RNAP II association with the different regions of *RPB2* gene in cells expressing a truncated Spt5 were normalized to those in cells expressing the full-length wild type Spt5. The Student's *t*-test was used for statistical analysis.

### Repair analysis of UVC-induced CPDs

Yeast cells were grown at 30°C in SD medium to late log phase (*A*_600_ ≈ 1.0), irradiated with 120 J/m^2^ of 254 nm UV (from a 15W UV germicidal bulb, General Electric) and incubated in YPD medium in the dark at 30°C. At different times of the repair incubation, aliquots were removed and the genomic DNA was isolated using a hot SDS procedure as described previously (21).

The TS of *RPB2* gene were 3′ end labeled with [α-^32^P]dATP using a procedure described previously (29,30). Briefly, ∼1 μg of total genomic DNA was digested with DraI to release the *RPB2* fragment and incised at CPD sites with an excess amount of T4 endonuclease V. Excess copies of a biotinylated oligonucleotide, which is complementary to the 3′ end of the TS of *RPB2*, were mixed with the samples. The mixtures were heated at 95°C for 5 min to denature the DNA and then cooled to an annealing temperature of around 50°C. The annealed molecules were attached to streptavidin magnetic beads, labeled with [α-^32^P]dATP, and resolved on DNA sequencing gels. The gels were exposed to a Phosphorimager screen. The intensities of gel bands corresponding to CPD sites were quantified using Quantity One software (Bio-Rad).

## RESULTS

### Spt5 interacts with the clamp, protrusion and wall domains of RNAP II

Previous structural and biochemical studies have shown that the NGN domain of archaeal Spt5 directly interacts with the coiled coil of RNAP clamp, and may have close proximity to the protrusion and lobe domains (7–9,31). To determine if eukaryotic Spt5 interacts with RNAP II in a similar way, we used an *in vivo* site-specific cross-linking technique (32,33). This technique utilizes a pair of plasmids to specifically substitute a residue of a protein of interest with Bpa, a photoreactive unnatural amino acid (Supplementary Figure S2A–C). Upon irradiation with UVA (350–365 nm), Bpa can react with another carbon within a short distance of approximately 3 angstroms (34). In contrast to many traditional methods, which cannot distinguish direct and indirect interactions, this technique allows unambiguous detection of direct protein–protein interactions in living cells, as only if a Bpa has a direct contact with an interacting partner can a cross-link be induced by UVA irradiation. Indeed, Bpa substitution of Rpb7 F42, which is known to interact with Rpb4, cross-linked to Rpb4 (Supplementary Figure S2D and E). However, no cross-linking between Rpb4 and Rpb7 can be detected if Bpa substituted a residue on the surface of Rpb7 that does not contact with Rpb4 (data not shown).

**Figure 1.**
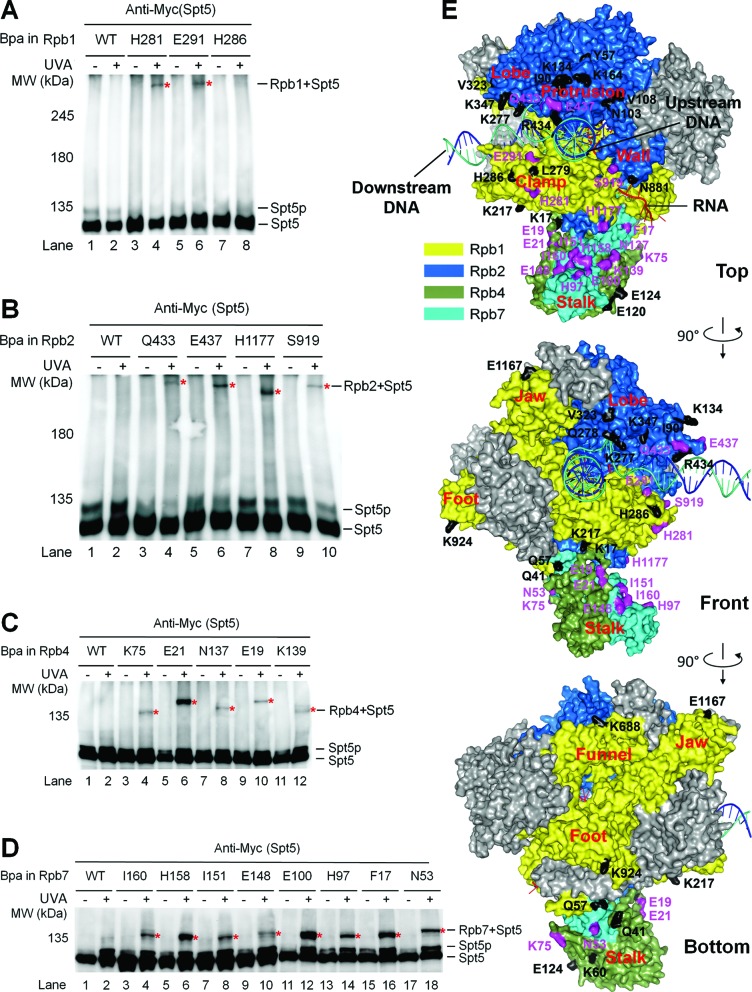
Spt5 interacts with the clamp, protrusion, wall and Rpb4/7 stalk of RNAP II. (**A**–**D**) Western blots showing cross-linking of Bpa-substituted Rpb1, Rpb2, Rpb4 and Rpb7 to Spt5. 3×Myc-tagged Spt5 was detected with an anti-Myc antibody on the western blots. Sites of Bpa substitutions are shown above the lanes of each blot. Bands of Spt5 cross-linked to Bpa-substituted proteins are marked with red asterisks. Spt5p stands for phosphorylated Spt5, which disappears following phosphatase treatment or deletion of Spt5 CTR or the *BUR2* gene (23). (**E**) Locations of Bpa-substituted residues on the surface of RNAP II. Rpb1, Rbp2, Rbp4 and Rpb7 are shown in colors as indicated. The other subunits of RNAP II are shown in gray. The template DNA, non-template DNA, and RNA transcript are shown as blue, greencyan and red ribbon structures, respectively. Residues that cross-linked to Spt5 are shown in purple and those that did not cross-link to Spt5 are shown in black. Bpa substitution at Rpb2 Y931, which did not cross-link to Spt5, is not shown, as this site is missing in the crystal structure. The RNAP II structure is based on PDB 1Y1W (35), which, in addition to the protein components, reveals the locations of downstream DNA and the DNA–RNA hybrid in the transcription bubble. The locations of the non-template DNA strand in the transcription bubble and the upstream DNA duplex are based on single molecule fluorescence resonance energy transfer studies and modeling (9,36). See Supplementary Tables S4–S7 for lists of all Bpa-substituted residues in Rpb1, Rpb2, Rpb4 and Rpb7.

We created yeast cells expressing 3×Myc-tagged Spt5 and Bpa-substituted Rpb1 or Rpb2 (Supplementary Tables S4 and S5). The yeast cells were cultured in a medium containing Bpa. Whole cell extracts were prepared directly from the cells or following irradiation of the cells with UVA. The proteins were resolved on SDS-PAGE, which disrupts non-covalent protein–protein interactions and separates proteins primarily based on protein sizes, and subjected to Western blot. Bpa substitutions at Rpb1 H281 and E291, located at the coiled coil of the RNAP II clamp, caused slower migrating bands of Spt5 upon UVA irradiation (Figure 1A and E, Supplementary Figure S3A), reflecting cross-linking of Rpb1 to Spt5 at these sites. Among the Bpa substitutions of Rpb2 residues, three located at the RNAP II protrusion (K426, F429 and R430) were found to be lethal (Supplementary Table S5). Viable Bpa substitutions located at the tip of the protrusion (Q433, E437), the base of the clamp (H1177) and the region of the wall (S919) that is adjacent to the clamp cross-linked to Spt5 (Figure 1B and E, Supplementary Figure S3B). Note that two proteins cross-linked at different sites may migrate differently on the gel. These results indicate that Spt5 interacts with the clamp, protrusion and wall domains of RNAP II in yeast.

### Spt5 also extensively interacts with Rpb4/7

The Rpb4/7 subcomplex forms the peripheral stalk structure of RNAP II (37,38). Deletion of Rpb4 or Spt4 was shown to restore TCR in *rad26Δ* cells, indicating that these factors repress TCR in the absence of Rad26 (21,22). We later found that the role of Spt4 in repressing TCR is indirect by protecting Spt5 from degradation and stabilizing the interaction of Spt5 with RNAP II (23). However, if and/or how Spt5 and Rpb4 coordinate in repressing TCR has been unclear. We therefore determined if Spt5 and Rpb4/7 physically interact. Indeed, Bpa substitutions at multiple sites of Rpb4 and Rpb7 caused slower migrating bands of Spt5 upon UVA irradiation (Figure 1C–E, Supplementary Tables S6 and S7), reflecting cross-linking of Rpb4 and Rpb7 to Spt5 at these sites. It is intriguing to note that the sites of Rpb4 and Rpb7 that cross-link to Spt5 are distributed almost all around the cylindrical surface of the stalk structure. These results indicate that Spt5 interacts with Rpb4 and Rpb7 so extensively that it may actually wrap around the stalk structure of RNAP II. However, as transcription elongation is a dynamic process, the interactions between Spt5 and Rpb4/7 may not reflect a single static conformation in the Spt5-RNAP II complex.

Upon UVA irradiation, Bpa-substituted Rpb4 and Rpb7 showed multiple slower migrating bands on Western blots (Supplementary Figure S3C and D). This indicates that Rpb4/7 directly interacts with other proteins in addition to Spt5. This is consistent with the fact that Rpb4/7 also interacts with various transcription factors during transcription initiation and executes some non-transcriptional activities, including mRNA transport (39).

### Domains of Spt5 that interact with RNAP II and a model of Spt4/5-RNAP II interaction architecture

**Figure 2.**
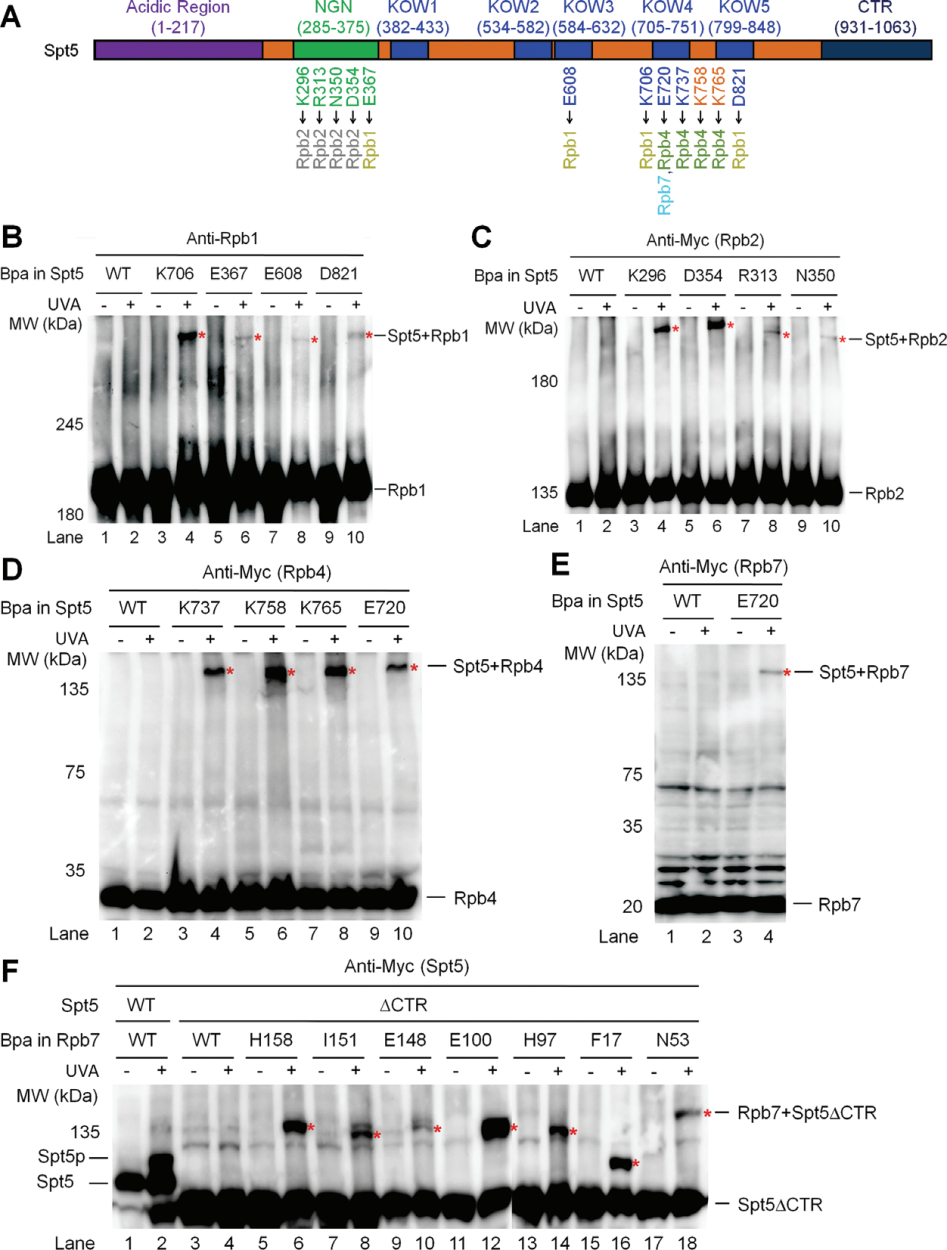
Domains of Spt5 that interact with RNAP II. (**A**) Schematic showing different domains of the yeast Spt5. Spt5 residues that cross-link to Rpb1, Rpb2, Rpb4 and Rpb7 are shown below the schematic. (**B**–**E**) Western blots showing cross-linking of Bpa-substituted Spt5 to Rpb1, Rpb2, Rpb4 and Rpb7. Rpb1 was detected with antibody 8WG16. 3×Myc-tagged Rpb2, Rpb4 and Rpb7 were detected with an anti-Myc antibody. Sites of Bpa substitutions are shown above the lanes of each blot. Bands of cross-linked proteins are indicated with red asterisks. (**F**) Western blot showing cross-linking of Bpa-substituted Rpb7 to CTR-deleted Spt5 (Spt5ΔCTR). Spt5p stands for phosphorylated Spt5, which disappears following phosphatase treatment or deletion of Spt5 CTR or the *BUR2* gene (23). 3×Myc-tagged Spt5 was detected with an anti-Myc antibody.

To date, only the crystal structure of the yeast Spt5 NGN domain bound to Spt4 has been reported (10). To gain insights into the architecture of Spt5-RNAP II interactions, we generated model structures of Spt5 using I-TASSER (40) (Supplementary Figure S4). The model structures of Spt5 may be disparate from real situation because even the NGN domain of Spt5 in the model structures is very different from that in the real crystal structure of Spt5 NGN bound to Spt4 (10). However, the model structures provided us with certain guidance on the likely interface residues of Spt5. We substituted Bpa for Spt5 residues that are likely to be involved in interactions with other proteins (Supplementary Figure S4 and Table S8). Bpa substitutions at Spt5 E367 (NGN), E608 (KOW3), K706 (KOW4) and D821 (KOW5) cross-linked to Rpb1 (Figure 2A and B, Supplementary Figure S5A). Bpa substitutions at Spt5 K296, R313, N350 and D354, all located in the NGN domain, cross-linked to Rpb2 (Figure 2A and C, Supplementary Figure S5B). Furthermore, Bpa substitutions at Spt5 E720, K737, K758 and K765, located in the KOW4 and KOW4-5 linker regions, cross-linked to Rpb4 (Figure 2A and D, Supplementary Figure S5C). Interestingly, Bpa substitution at Spt5 E720 also cross-linked to Rpb7 (Figure 2A and E, Supplementary Figure S5D), indicating that this Spt5 residue is located at the boundary between Rpb4 and Rpb7.

Like Rpb4, the CTR domain of Spt5, which can be phosphorylated by the Bur1/2 kinase complex, plays an important role in repression of TCR (23). We wondered if the Spt5 CTR interacts with Rpb4/7. However, Bpa substitution of residue Y1011 in the Spt5 CTR domain did not cross-link to Rpb4/7 (Supplementary Table S8). We then tested cross-linking of Rpb7 to the CTR-deleted Spt5 (residues 1–870 remaining). Bpa substitution of Rpb7 I160 is lethal when the Spt5 CTR is deleted (Supplementary Table S9). This lethality may be due to the combination of the Bpa substitution and Spt5 CTR deletion, as this substitution is viable when the full length Spt5 is present (Supplementary Table S7). As expected, phosphorylation can be detected in the wild type Spt5 but not the CTR-deleted Spt5 (Spt5ΔCTR) (Figure 2F). All the viable Bpa substitutions of Rpb7 residues normally cross-linked to the CTR-deleted Spt5 (compare Figure 2F with Figure 1D). These results do not support a direct interaction between the Spt5 CTR and RNAP II. This is in agreement with previous studies showing that the CTR domain of Spt5 in human (41,42) or yeast (23,43) cells is not required for binding of Spt5 to RNAP II.

**Figure 3.**
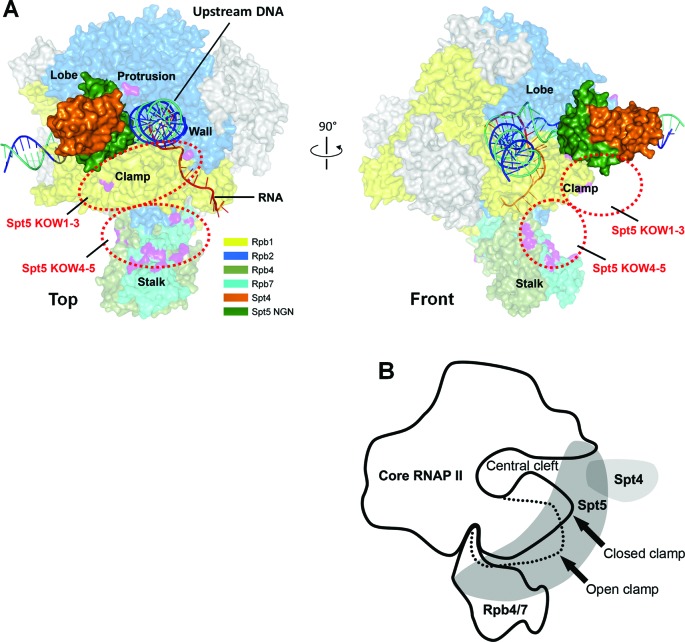
Architecture of interactions between Spt4/5 and RNAP II. (**A**) Locations of Spt4 and different domains of Spt5 on RNAP II. The sources of the RNAP II structure are the same as those of Figure 1E. Spt4, the NGN domain of Spt5, Rpb1, Rbp2, Rbp4 and Rpb7 are shown in colors as indicated. Other subunits of RNAP II are shown in gray. The template DNA, non-template DNA and RNA transcript are shown as blue, greencyan and red ribbon structures, respectively. Residues of RNAP II that cross-linked to Spt5 are shown in purple. The Spt4/Spt5-NGN structure [PDB 2EXU (10)] is docked onto the RNAP II clamp based on the archaeal Spt4/5-RNAP clamp structure (9) and our Bpa cross-linking data. The likely locations of the Spt5 KOW domains are marked with red dashed-line ellipses. (**B**) Schematic cut-away view of the interaction architecture. The dashed line indicates the open clamp position observed in the absence of Rpb4/7 [PDB 1I50 (44)].

In view of the observations that the archaeal Spt5 NGN interacts with the RNAP clamp and has close proximity to the protrusion (9), it is highly likely that the yeast Spt5 NGN interacts with Rpb1 through the clamp domain and with Rpb2 through the protrusion domain. Spt4 may bind to the other side of the Spt5 NGN and point away from RNAP II (Figure 3A and B). Based on our findings that the Spt5 KOW4 interacts with Rpb1, Rpb4 and Rpb7 (Supplementary Table S8), this domain is likely to reside in the indentation between the Rpb1 clamp and the Rpb4/7 stalk. The Spt5 KOW5 may also reside in or be close to the indentation, as this domain cross-links to Rpb1 and the KOW4-5 linker cross-links to Rpb4. The Spt5 KOW3 may interact with the clamp region that is between the coiled coil and the base of the clamp. The KOW1, which is adjacent to the NGN, and KOW2, which is adjacent to KOW3, may also reside in or be close to the region between the coiled coil and the base of the clamp, although we did not detect direct interactions of the KOW1-2 with any RNAP II subunits. It is possible that the KOW1–2 domains of Spt5 bulge away from the RNAP II surface. The acidic and CTR domains of Spt5 might not directly interact with RNAP II. This is supported by previous studies showing that the acidic and CTR domains of Spt5 are not required for binding of Spt5 to RNAP II (23,41-43). We must note that the proposed model of Spt4/5-RNAP II interaction architecture is based on non-exhaustive Bpa substitutions.

We attempted to dock model structures of different Spt5 KOW domains [obtained by using the I-TASSER server (40)] onto the crystal structures of elongating RNAP II by using multiple pieces of docking software. However, none of the docking results appeared to be reasonable, including those of Spt5 KOW3, KOW4, KOW5, KOW3–4 or KOW4-5, which we found directly interact with Rpb1, Rpb4 and Rpb7. This can be due to the following: (i) the model structures are not accurate enough and/or (ii) the crystal structures of RNAP II may be somewhat different from the dynamic structures of the enzyme in the cell.

### Deletion of Spt5 KOW4 or KOW4-5 domains decreases transcription elongation

**Figure 4.**
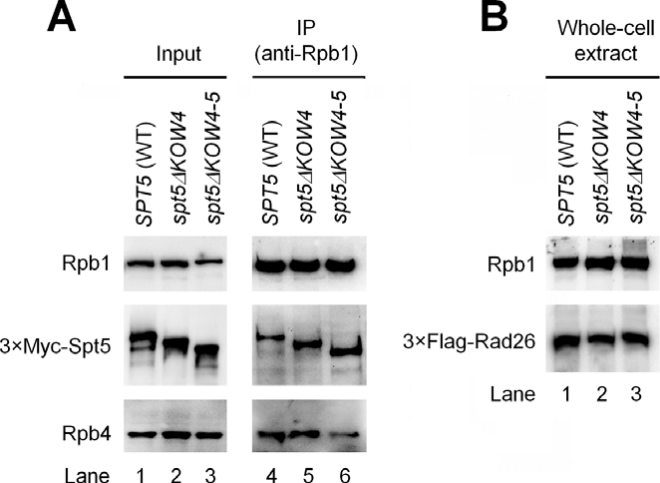
Effects of Spt5 KOW4 and KOW4-5 deletions on the association of Rpb4 with core RNAP II and the cellular levels of Rad26. (**A**) Deletion of Spt5 KOW4-5, but not KOW4, reduced co-immunoprecipitation of Rpb4 with core RNAP II. Western blots show the levels of Rpb1, Rpb4 and 3×Myc-tagged Spt5 in chromatin fractions of whole cell extracts (input) and in samples immunoprecipitated (IP) by using the anti-Rpb1 antibody 8WG16. (**B**) Deletion of Spt5 KOW4 or KOW4-5 did not significantly affect the cellular levels of Rad26. Rpb1, Rpb4, 3×Myc-tagged Spt5 and 3×Flag-tagged Rad26 were detected with 8WG16, 2Y14, anti-Myc and anti-Flag antibodies, respectively, on the blots. The western blots shown are representatives from three experiments.

The Rpb4/7 stalk is easily dissociable from the 10 subunit core RNAP II *in vitro* (37,38). Association of the Rpb4/7 stalk with the core RNAP II ‘wedges’ the clamp to the closed conformation, resulting in a narrower central cleft of the polymerase (Figure 3B) (37,38). The interactions of Spt5 with the Rpb4/7 stalk, clamp and protrusion of RNAP II may lock the clamp in the closed conformation and enclose the DNA being transcribed in the central cleft of the polymerase, thereby enhancing transcription elongation. To test this idea, we deleted Spt5 KOW4 (residues 706–765 removed) and KOW4-5 (residues 706–848 removed), which are involved in the interaction with Rpb4/7 (Supplementary Table S8). These deletions, especially the KOW4-5 deletion, are expected to disrupt the interactions between Spt5 and Rpb4/7. Deletion of KOW4 or KOW4-5 did not reduce the expression of Spt5 or co-immunoprecipitation of Spt5 with RNAP II (Figure 4A). However, the KOW4-5 deletion caused dramatic (over 2 fold) reduction of Rpb4 co-immunoprecipited with RNAP II (Figure 4A), indicating that the KOW4-5 domains of Spt5 stabilize the interaction of the Rpb4/7 stalk with the core RNAP II.

**Figure 5.**
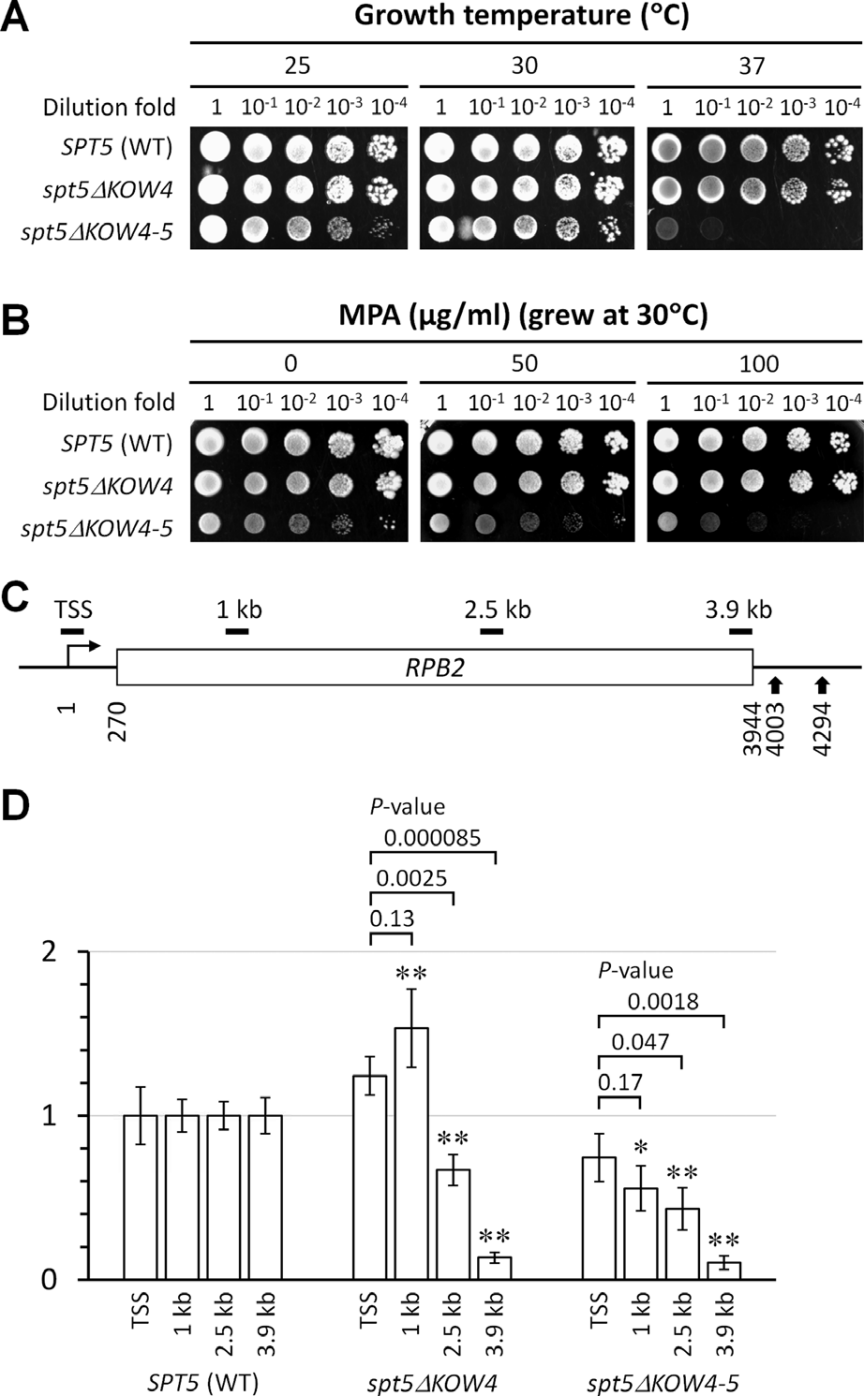
Deletion of Spt5 KOW4-5 causes defects in transcription elongation. (**A**) Growth of yeast cells expressing wild type (WT), KOW4 deleted (*spt5ΔKOW4*) and KOW4-5 deleted (*spt5ΔKOW4-5*) Spt5 at different temperatures. (**B**) Growth of the different yeast strains in the presence of different concentrations of mycophenolic acid (MPA), a nucleotide depletion drug. (**C**) Schematic of the *RPB2* gene. Nucleotide positions are relative to the transcription start site (TSS). Vertical arrows at the 3′ end of the gene indicate the two alternative polyadenylation sites (48). Short horizontal bars above the schematic indicate regions of 134–150 bp amplified by real-time PCR for quantification of ChIP fragments of the *RPB2* gene (see Supplementary Table S3). (**D**) RNAP II densities in different regions of the *RPB2* gene. The RNAP II densities in the TSS, 1 kb, 2.5 kb and 3.9 kb regions of the *RPB2* gene in WT cells were normalized to 1. The RNAP II densities in the different regions of the *RPB2* gene in *spt5ΔKOW4* and *spt5ΔKOW4-5* cells are relative to those in the corresponding regions of the *RPB2* gene in WT cells. The values of RNAP II densities are represented as mean (+/− S.D.) of three ChIP experiments. Single asterisk (*) and double asterisks (**) denote a *P*-value of <0.05 and <0.01, respectively, in the Student's *t*-test between the mutant and WT cells for RNAP II densities in the corresponding regions of the *RPB2* gene. The RNAP II densities in the TSS region of the *RPB2* gene in *spt5ΔKOW4* and *spt5ΔKOW4-5* cells were not significantly different from that in the WT cells (*P*-values are 0.1 and 0.3 for the *spt5ΔKOW4* and *spt5ΔKOW4-5* cells, respectively; a *P*-value of <0.05 is considered to be significant). Above the bars of *spt5ΔKOW4* and *spt5ΔKOW4-5* samples are shown the *P*-values of Student's *t*-test between the TSS region and the 1, 2.5 or 3.9 kb region.

While Spt5 KOW4 deleted (*spt5ΔKOW4*) cells grew almost normally, Spt5 KOW4-5 deleted (*spt5ΔKOW4-5*) cells had growth defects especially at an elevated temperature (37°), suggesting a defect in transcription elongation (Figure 5A). The temperature sensitivity of *spt5ΔKOW4-5* cells is similar to that of *rpb4Δ* cells (45). *spt5ΔKOW4-5* cells are also somewhat more sensitive to the nucleotide depletion drug MPA than wild type cells (Figure 5B). Sensitivity to a nucleotide depletion drug is often correlated with an elongation defect, although the underlying mechanism can be more complicated (46). The drug sensitivity of *spt5ΔKOW4-5* cells is also similar to that of *rpb4Δ* cells (47), indicating that Spt5 and Rpb4 may coordinate to ensure efficient transcription elongation, especially at an elevated temperature.

We then measured the densities of RNAP II in different regions of a transcribed gene by using the ChIP assay. Sonicated chromatin fragments (200 bp on average) were immunoprecipitated with antibody 8WG16, which recognizes the CTRs of Rpb1, the largest subunit of RNAP II. The immunoprecipitated fragments located at different regions of the *RPB2* gene were quantified by using real-time PCR. The RNAP II densities in different regions of the *RPB2* gene in wild type cells were normalized to 1 and those in *spt5ΔKOW4* and *spt5ΔKOW4-5* cells were represented as values relative to the wild type cells (Figure 5C and D). The reason for us to choose the *RPB2* gene for analysis is that we have extensively studied TCR in this RNAP II transcribed housekeeping gene (see below). The RNAP II densities in the transcription start site (TSS) in *spt5ΔKOW4* and *spt5ΔKOW4-5* cells were not significantly different from that in wild type cells (Figure 5D), indicating that these mutant cells have relatively normal transcription initiation. However, for an unknown reason, the RNAP II density in the 1 kb region was higher in *spt5ΔKOW4* cells than in wild type cells, which may compensate for the deficiency in transcription elongation and explain why the *spt5ΔKOW4* cells grew almost normally and were resistant to MPA (Figure 5A and B). Compared to wild type cells, both *spt5ΔKOW4* and *spt5ΔKOW4-5* cells showed a gradual decrease in RNAP II densities toward the 3′ end of the *RPB2* gene (Figure 5D). It is quite unlikely that the gradual decrease of RNAP II density toward the 3′ end of the *RPB2* gene in the *spt5ΔKOW4* and *spt5ΔKOW4-5* cells is caused by a gradual increase in transcription elongation rate toward the 3′ end, or a gradual decrease of transcription elongation rate toward the 5′ end of the gene. Rather, the gradual decrease of RNAP II density can be caused by a deficiency in transcription processivity in the absence of Spt5 KOW4 or KOW4-5.

### Deletion of Spt5 KOW4-5 derepresses TCR

**Figure 6.**
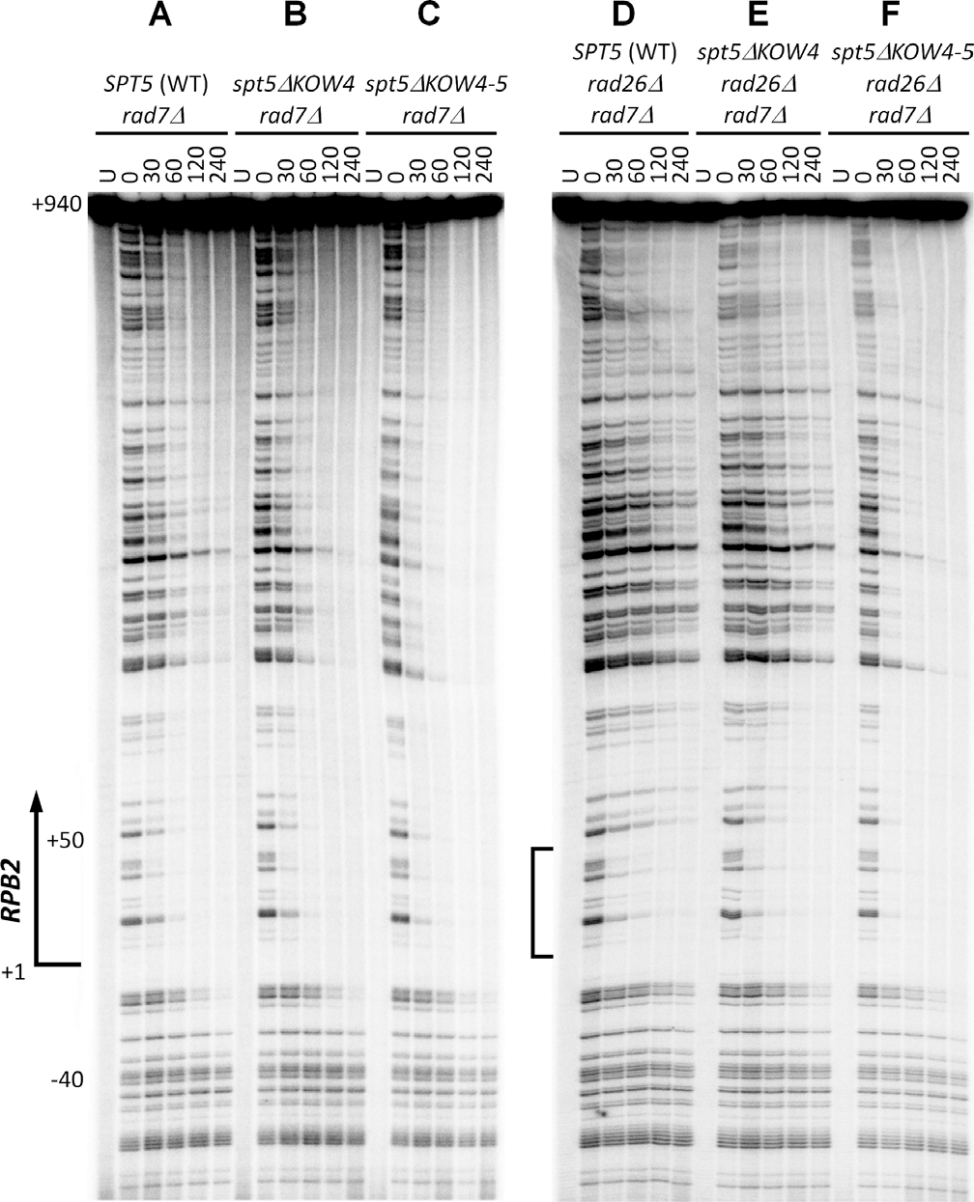
Deletion of Spt5 KOW4-5 derepresses TCR. (**A**–**C**) Sequencing gel showing TCR of CPDs in *rad7Δ* cells expressing wild type (WT), KOW4 deleted (*spt5ΔKOW4*) and KOW4-5 deleted (*spt5ΔKOW4-5*) Spt5. Unirradiated (U) and irradiated samples after different times (in minutes) of repair incubation are indicated at the top of the gel lanes. Nucleotide positions shown on the left are relative to the TSS. (**D**–**F**) Sequencing gel showing TCR of CPDs in *rad7Δ rad26Δ* cells expressing wild type, KOW4 deleted and KOW4-5 deleted Spt5. Bracket on the left of panel (D) indicates the region immediately downstream of the TSS where TCR is not significantly repressed even in the absence of Rad26.

**Figure 7.**
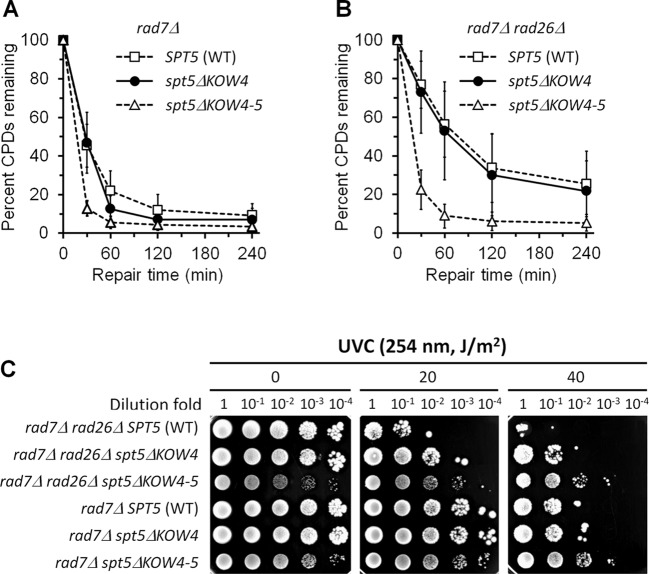
Effects of Spt5 KOW4 and KOW4-5 deletions on TCR. (**A** and **B**) Percent of CPDs remaining in the TS of the *RPB2* gene in *rad7Δ* and *rad7Δ rad26Δ* cells expressing wild type (WT), KOW4 deleted (*spt5ΔKOW4*) and KOW4-5 deleted (*spt5ΔKOW4-5*) Spt5. Data are represented as mean +/− S.D. (**C**) Survival of *rad7Δ* and *rad7Δ rad26Δ* cells expressing wild type, KOW4 deleted and KOW4-5 deleted Spt5 following different doses of UVC irradiation.

The closed conformation of RNAP II, which can be stabilized by Spt5 and is highly competent for transcription elongation, may trap a DNA lesion in the central cleft and repress TCR. If this is the case, disruption of the interactions between Spt5 and the Rpb4/7 stalk may derepress TCR. To test this idea, we analyzed the effects of Spt5 KOW4 and KOW4-5 deletions on TCR. In yeast, Rad7 and its interaction partner Rad16 are essential for global genomic repair (GGR) but play no role in TCR (49). Therefore, TCR can be unambiguously analyzed in *rad7Δ* or *rad16Δ* cells. A nucleotide resolution method that uses streptavidin magnetic beads and biotinylated oligonucleotides to facilitate isolation and strand-specific end-labeling of DNA fragments of interest was used for the analysis (29,30). TCR, which initiates about 40 nucleotides upstream of the TSS of the *RPB2* gene, could be seen in *rad7Δ* cells (Figure 6A). As expected, the additional deletion of *RAD26* (*rad7Δ rad26Δ*) decreased TCR in the transcribed region of the gene (Figure 6, compare panels A and D), except for a region of ∼50 nucleotides immediately downstream of the TSS (Figure 6D, marked with the bracket). Our results agree with previous studies showing that TCR in a short region (20–50 nucleotide long) immediately downstream of the TSS of a gene is rapid and less dependent on Rad26 in yeast (21,50) or CSA and CSB in mammalian (51,52) cells, indicating that the short region is less repressed even in the absence of Rad26, CSA or CSB. While the Spt5 KOW4 deletion slightly enhanced TCR, the Spt5 KOW4-5 deletion dramatically enhanced the repair event throughout the transcribed region of the *RPB2* gene in both *rad7Δ* and *rad7Δ rad26Δ* cells (Figure 6, compare panels B and C with A, and E and F with D; Figure 7A and B). Note that the TCR speed in *rad7Δ rad26Δ spt5ΔKOW4-5* cells was even faster than that in *rad7Δ* (*RAD26^+^* and *SPT5^+^*) cells, especially in the region ∼50 nucleotides downstream of the TSS (Figure 6, compare panels A and F; Figure 7A and B). The enhancement of TCR by deletion of the Spt5 KOW4-5 is similar to that by deletion of Rpb4 (21) but more dramatic than that by deletion of Spt4 (22), the Spt5 CTR (23) or subunits of PAFc (24). Also, in contrast to deletions of Spt4, the Spt5 CTR and subunits of PAFc, which enhance TCR only in the absence but not in the presence of Rad26, deletion of the Spt5 KOW4-5 (or Rpb4) enhances TCR in the absence or presence of Rad26. The dramatic enhancement of TCR in *spt5ΔKOW4-5* cells is not due to a change in Rad26 levels in the cell, as deletion of Spt5 KOW4 or KOW4-5 did not significantly affect the cellular levels of Rad26 (Figure 4B). Rather, it is likely that the full-length Spt5, by coordinating with Rpb4/7, strongly represses TCR and Rad26 can only partially antagonize its repression effect. On the other hand, Rad26 appears to be able to completely antagonize the repression effects of Spt4, the Spt5 CTR and PAFc. Spt5 (along with Spt4) is loaded to RNAP II by binding to the nascent transcript only after it reaches to 30–50 nucleotides in length (53), which may explain why TCR in the short region immediately downstream of the TSS is not significantly repressed by Spt5.

In agreement with the enhancement of TCR, deletion of Spt5 KOW4 or KOW4-5 in *rad7Δ* or *rad7Δ rad26Δ* cells enhanced survival of yeast cells upon UVC (254 nm) irradiation, with the KOW4-5 deletion being more striking (Figure 7C). Also, in agreement with their highly efficient TCR, *rad7Δ rad26Δ spt5ΔKOW4-5* and *rad7Δ spt5ΔKOW4-5* cells were more UVC resistant than *rad7Δ* (*RAD26^+^* and *SPT5^+^*) cells (Figure 7C). Taken together, our results indicate that the Spt5 KOW4-5 domains play a pivotal role in repression of TCR even in the presence of Rad26.

### TFIIE interacts with the clamp and Rpb4/7 stalk of RNAP II differently from Spt5, which may explain why TFIIE does not significantly repress TCR in the short region immediately downstream of the transcription start site

**Figure 8.**
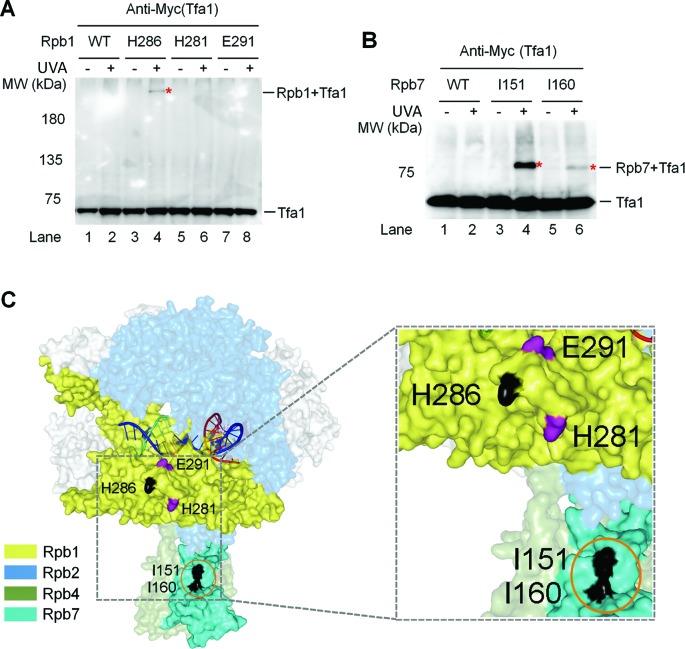
TFIIE and Spt5 interact with the clamp and Rpb4/7 stalk of RNAP II differently. (**A** and **B**) Western blots showing cross-linking of Bpa-substituted Rpb1 and Rpb7 to Tfa1, respectively. 3×Myc-tagged Tfa1 was detected with an anti-Myc antibody. Sites of Bpa substitutions are shown above the lanes of each blot. Bands of Tfa1 cross-linked to Bpa-substituted Rpb1 and Rpb7 are indicated with red asterisks. (**C**) Locations of Bpa-substituted Rpb1 and Rpb7 residues that cross-linked to Tfa1. Residues that cross-linked to Tfa1 are shown in black. Orange circle indicates Rpb7 residues that also cross-linked to Spt5 (see Figure 1D and E). The two Rpb1 residues (E291 and H281) that cross-linked to Spt5 (see Figure 1A and E) but not Tfa1 are shown in purple. The RNAP II structure is based on PDB 1Y1W (35).

Förster resonance energy transfer analysis showed that the archaeal transcription initiation factor TFE, the homolog of eukaryotic TFIIE alpha subunit (Tfa1), and Spt4/5 compete for binding to the RNAP clamp during the transition from transcription initiation to elongation (31). Studies with hydroxyl radical-generating probes (54) and cryoelectron microscopy (55,56) have also shown that the yeast Tfa1 interacts with the RNAP II clamp and extends to the Rpb4/7 stalk of RNAP II. Therefore, TFIIE and Spt5 may employ a similar binding strategy to enclose the DNA in the central cleft to facilitate transcription initiation and elongation, respectively. However, why is TCR in the short region immediately downstream of the TSS of a gene not significantly repressed by TFIIE? One possibility is that the interactions of RNAP II with TFIIE during transcription initiation and those with Spt5 during transcription elongation may be different. To test this idea, we mapped the sites of RNAP II that interact with Tfa1. Indeed, Bpa substitution at Rpb1 H286, located on the RNAP II clamp, cross-linked to Tfa1 (Figure 8A and C). However, this site did not cross-link to Spt5 (Figure 1A and E). On the other hand, Bpa substitutions at Rpb1 H281 and E291, which are close to H286 on the clamp, cross-linked to Spt5 (Figure 1A and E) but not Tfa1 (Figure 8A and C, Supplementary Table S4). Bpa substitutions at Rpb7 I151 and I160, which cross-link to Spt5 (Figure 1D and E), also cross-linked to Tfa1 (Figure 8B and C). However, Bpa substitutions at Rpb7 F17, N53, H97, E100, E148 and H158, which cross-link to Spt5, did not cross-link to Tfa1 (Supplementary Table S7). Therefore, the sites of RNAP II clamp and Rpb4/7 stalk that interact with Spt5 partially overlap with those that interact with TFIIE. This finding is consistent with the ‘factor swapping’ mechanism that has been proposed to explain the transition from RNAP II initiation to elongation (3). As described above, Spt5 may wrap around the Rpb4/7 stalk, which may greatly stabilize the interaction of the stalk to the core RNAP II. The different patterns of interactions of TFIIE with RNAP II may explain why TCR is not repressed by the transcription initiation factor in the region immediately downstream of the TSS.

## DISCUSSION

### Spt5 is a key transcription elongation factor

Our mapping of the interactions of Spt5 with RNAP II provides insights into the functional mechanisms of this transcription elongation factor. Although the Rpb4/7 stalk is easily dissociable from the 10 subunit core RNAP II and is not essential for transcription elongation *in vitro*, Rpb4 and Rpb7 play important roles throughout the transcription cycle *in vivo* (47,57). Crystal structures of the 12 subunit yeast RNAP II show that Rpb4/7 interacts with the 10 subunit core RNAP II through a small ‘tip’ of the Rpb7 wedge structure and most of the Rpb4/7 surface is not involved in the interaction (37,38). Except for wedging the RNAP II clamp to the closed conformation, association of Rpb4/7 does not cause a gross change in the structure of the core RNAP II. The interactions of Spt5 with the clamp, protrusion, wall and Rpb4/7 should stabilize the association of Rpb4/7 with the core RNAP II, thereby locking the clamp in the closed conformation and enclosing the DNA being transcribed in the central cleft of the polymerase to facilitate transcription processivity (Figure 3). In addition, Spt5 may directly interact with the upstream DNA and the Spt5 KOW4-5 domains may have close proximity to the RNA exiting channel and interact with the nascent transcript (4). These interactions may also facilitate transcription elongation by repressing transcription pausing and backtracking (4).

Unlike Spt5, which is essential for cell viability, the small zinc finger protein Spt4 is dispensable for cell survival. Spt4 binds to the NGN domain of Spt5 but may not directly interact with RNAP II (9,10). Spt4 protects Spt5 from degradation and stabilizes the binding of Spt5 to RNAP II (23). PAFc, which is also not essential for cell viability, is recruited to RNAP II complex through interaction with the CTR domain of Spt5 (24,58-60). It is therefore likely that, through direct interactions with the clamp, protrusion, wall and Rpb4/7 stalk of RNAP II, Spt5 serves as a key transcription elongation factor. On the other hand, Spt4 and PAFc may serve as accessory transcription elongation factors by interacting with the Spt5 NGN and CTR domains, respectively.

Similar to eukaryotic Spt5, archaeal Spt5 (7–9) and bacterial NusG (61) also bind to the clamp of an RNAP and may enclose the DNA being transcribed in the central cleft. However, the functional mechanism of a eukaryotic Spt5 is likely to be significantly different from that of an archaeal Spt5 or a bacterial NusG. We show here that the KOW4-5 domains of the yeast Spt5 extensively interact with Rpb4/7 and deletion of these domains significantly decreases transcription elongation. However, archaeal Spt5 or bacterial NusG has a single KOW domain and a bacterial RNAP lacks the eukaryotic Rpb4/7 counterparts. The single KOW domain of an archaeal Spt5 may not be able to reach and extensively interact with RpoF/E, the counterpart of the eukaryotic Rpb4/7. Therefore, although Spt5 and RpoF/E play important roles in transcription elongation in archaea (62), an archaeal RNAP elongation complex may not be stabilized primarily by interactions between Spt5 and RpoF/E. In *E. coli*, the single KOW domain of NusG may interact with other transcription regulators, rather than with a subunit of RNAP (61,63).

### Spt5 is a key TCR repressor

DNA lesions that are NER substrates are stalled at the active site of RNAP II following incorporation or misincorporation of nucleotide(s) opposite to the damaged template (64–66). Our finding that Spt5 interacts with the clamp, protrusion, wall and Rpb4/7 stalk of RNAP II suggests that a DNA lesion can be trapped in the closed elongation complex, rendering it inaccessible to the repair machinery.

We found that deletion of Spt5 KOW4-5 domains, which extensively interact with Rpb4/7, enhances TCR in *rad26Δ* and *RAD26^+^* cells. Similarly, deletion of Rpb4 also enhances TCR in *rad26Δ* and *RAD26^+^* cells (21). In contrast, deletion of Spt4 or subunits of PAFc enhances TCR to a lesser extent and the enhancement of TCR can only be seen in *rad26Δ* but not *RAD26*^+^ cells (22,24). These findings indicate that the full-length Spt5 and Rpb4 (in complex with Rpb7) are strong TCR repressors and they can repress TCR in the presence or absence of Rad26. On the other hand, Spt4 and PAFc are weaker TCR repressors and they can repress TCR only in the absence but not in the presence of Rad26. Therefore, Spt5, through direct interactions with Rpb4/7 and other domains of RNAP II, appears to play a key role in repressing TCR. Spt4 and PAFc, by interacting with the NGN and CTR domains of Spt5, respectively, may play accessory roles in repressing TCR by further stabilizing the closed elongation competent RNAP II complex. Rad26 appears to be able to completely antagonize the accessory TCR repressors but can only partially alleviate the repression of TCR by Spt5.

It has been shown recently that the *E. coli* UvrD induces backtracking of RNAP, and thereby exposing DNA lesions shielded by RNAP and allowing NER enzymes to gain access to lesion sites (16). In contrast to UvrD, *E. coli* NusG inhibits backtracking and accelerates pause-free transcription by promoting forward translocation of RNAP (67). Therefore, like eukaryotic Spt5, NusG may also repress TCR in *E. coli* and UvrD may antagonize the repression by counteracting NusG. However, in view of the fact that the *E. coli* RNAP has no Rpb4/7 counterparts and the single KOW domain of *E. coli* NusG does not directly interact with RNAP, the underlying mechanisms of TCR repression in *E. coli* and eukaryotes may be somewhat different. By binding to an upstream activating sequence, the transcription activator Fis stimulates transcription of a tRNA gene to an extremely high level and at the same time represses TCR in the gene, except for a short region immediately downstream of the TSS (68). However, the repression of TCR in *E. coli* is apparently caused by extremely high level of loading of RNAP rather than by stabilization of the transcription elongation complex. During very high level transcription in a tRNA gene in *E. coli*, an RNAP may arrive at the site of a downstream RNAP stalled at a lesion before the downstream RNAP can initiate or finish the TCR process, resulting in repression of TCR (69). It is also possible that UvrD may not be able to efficiently backtrack RNAP molecules densely distributed in highly transcribed genes in *E. coli*. In eukaryotic cells, however, the loading of RNAP II to a gene being transcribed does not appear to be able to reach a level that can repress TCR. The galactose-induced *GAL1–10* genes are among the most highly transcribed genes by RNAP II in yeast. However, TCR occurs very rapidly in the *GAL1–10* genes in *RAD26*^+^ or *rad26Δ* cells (21,70), indicating TCR is not significantly repressed in these highly transcribed genes even in the absence of Rad26. In contrast, TCR is much slower in the much more slowly transcribed *RPB2* and *URA3* genes in *rad26Δ* cells (21,50). However, deletion of Rpb4 or Spt4 in *rad26Δ* cells restores TCR in the *RPB2* and *URA3* genes (21,22). These studies indicate that TCR is more repressed in the slowly transcribed genes in the absence of Rad26. Therefore, the repression of TCR in eukaryotic cells, which can be easily seen in *rad26Δ* cells, is not caused by extremely high level of loading of RNAP II but may be due to Spt5-coordinated stabilization of the transcription elongation complex. The reason why TCR is less repressed in the highly transcribed *GAL1–10* genes in *rad26Δ* cell is unknown, but may be due to a lower content of a TCR repressor in the RNAP complex engaged in highly transcribed genes.

## SUPPLEMENTARY DATA

Supplementary Data are available at NAR Online.

SUPPLEMENTARY DATA
