# Supplementary material for: Insights into how Spt5 functions in transcription elongation and repressing transcription coupled DNA repair
Source: Nucleic Acids Res. 2014 May 9;42(11):7069–83. doi: 10.1093/nar/gku333 (PMC4066765; doi:10.1093/nar/gku333)
Supplement: SUPPLEMENTARY DATA [file supp_42_11_7069__index.html]

SUPPLEMENTARY DATA 

# Insights into how Spt5 functions in transcription elongation and repressing transcription coupled DNA repair

## SUPPLEMENTARY DATA

**Files in this Data Supplement:**

- Supplementary material
