# Supplementary material for: Insights into how Spt5 functions in transcription elongation and repressing transcription coupled DNA repair
Source: Nucleic Acids Res. 2014 May 9;42(11):7069–83. doi: 10.1093/nar/gku333 (PMC4066765; doi:10.1093/nar/gku333)

SUPPLEMENTARY TABLES

Supplementary Table S1. Yeast strains used for shuffling with plasmids encoding Bpa-substituted proteins.

**Strain**[a]	**Genotype**[b]	**To be shuffled plasmids encoding Bpa substituted proteins**	**To be tested cross-linking partners**
WL119	phr1Δ rpb7Δ (RPB4-3×Myc) [pRS416-RPB7, pLH157]	Rpb7	Rpb4
WL120	phr1Δ rpb7Δ (SPT5-3×Myc) [pRS416-RPB7, pLH157]	Rpb7	Spt5
WL292	phr1Δ spt5Δ (RPB4-3×Myc) [pRS416-SPT5, pLH157]	Spt5	Rpb4
WL293	phr1Δ spt5Δ (RPB7-3×Myc) [pRS416-SPT5, pLH157]	Spt5	Rpb7
WL294	phr1Δ spt5Δ [pRS416-SPT5, pLH157]	Spt5	Rpb1
WL296	phr1Δ spt5Δ (RPB2-3×Myc) [pRS416-SPT5, pLH157]	Spt5	Rpb2
WL303	rpb7Δ spt5Δ [pRS416-RPB7, pNAT- SPT5ΔCTR, pLH157]	Rpb7	CTR deleted Spt5
WL491	phr1Δ rpb2Δ (SPT5-3×Myc) [pRS416-RPB2, pLH157]	Rpb2	Spt5
WL492	phr1Δ rpb1Δ (SPT5-3×Myc) [pRS416-RPB1, pLH157]	Rpb1	Spt5
WL543	phr1Δ rpb1Δ (TFA1-3×Myc) [pRS416-RPB1, pLH157]	Rpb1	Tfa1 (TFIIE)
WL590	phr1Δ rpb4Δ (SPT5-3×Myc) [pRS416-RPB4, pLH157]	Rpb4	Spt5
WL643	phr1Δ rpb7Δ (TFA1-3×Myc) [pRS416-RPB7, pLH157]	Rpb7	Tfa1 (TFIIE)

[a] All strains are isogenic to CR18 (*MATα ura3-52 trp1 his3 leu2 pep4::HIS3 rad7Δ rad26Δ*).
[b] Genomic genes tagged with 3×Myc are shown in parentheses; plasmids contained in a strain are shown in brackets.

**Supplementary Table S2.** SDS-PAGE conditions for Western-blot detection of cross-linkings between different proteins.			
**Bpa-substituted protein (kD)**	**Cross-linking partner (kD)**	**SDS-PAGE condition**	
		**For Western-blot of Bpa-substituted protein**	**For Western-blot of cross-linking partner**
Rpb1 (192)	Spt5 (116)	5-10%	5-10%
	Tfa1 (55)	5-10%	6-18%
Rpb2 (139)	Spt5 (116)	5-10%	5-10%
Rpb4 (25)	Spt5 (116)	6-18%	6-18%
Rpb7 (19)	Spt5 (116)	6-20%	6%
	Tfa1 (55)	10%	10%
	Rpb4 (25)	10%	10%
Spt5 (116)	Rpb1 (192)	5-12%	5-12%
	Rpb2 (139)	4-20%	4-20%
	Rpb4 (25)	8%	6-18%
	Rpb7 (19)	8%	6-18%

**Supplementary Table S3.** Primers used for real time PCR quantification of *RPB2* fragments immunoprecipitated by anti-Rpb1 antibody 8WG16.			
**Region**[a]	**Primer 1 (5' → 3')**	**Primer 2 (5' → 3')**	**Size of PCR product (bp)**
TSS	GGCGAACAAACAAGAAGT GAGT	ACCTGAGGAGAAGGAAT GAGTG	150
1 kb	AGGATATTCCTATTGTAAT CATATTC	AAACCCGTCTTCAACACA AG	134
2.5 kb	ATCATGCTACAACATTTAC ACATTGT	TAAAAACACACCCATAGC TTGC	149
3.9 kb	AACCAATTTGAATGTAAG GGA	AAAATCTCTCGAACGATC GGTA	141

[a] relative to the transcription start site (TSS).

**Supplementary Table S4.** Cross-linking of Bpa-substituted Rpb1 to Spt5 and Tfa1.				
**No.**	**Residue**	**Domain**	**Cross-linked to Spt5**	**Cross-linked toTfa1**
1	K217	Clamp head	No	x
2	L279	Clamp core	No	x
3	H281	Clamp core	Yes	No
4	H286	Clamp core	No	Yes
5	E291	Clamp core	Yes	No
6	K688	Funnel	No	x
7	K924	Foot	No	x
8	E1167	Jaw	No	x

"x" not tested.

**Supplementary Table S5.** Cross-linking of Bpa-substituted Rpb2 to Spt5.			
**No.**	**Residue**	**Domain**	**Cross-linked to Spt5**
1	Y57	Protrusion	No
2	I90	Protrusion	No
3	N103	Wall-Protrusion	No
4	V108	Wall-Protrusion	No
5	K134	Protrusion	No
6	K164	Protrusion	No
7	K277	Lobe	No
8	Q278	Lobe	No
9	V323	Lobe	No
10	K347	Lobe	No
11	K426	Protrusion	x
12	F429	Protrusion	x
13	R430	Protrusion	x
14	Q433	Protrusion	Yes
15	R434	Protrusion	No
16	E437	Protrusion	Yes
17	N881	Wall	No
18	S919	Wall	Yes
19	Y931	Wall	No
20	H1177	Clamp base	Yes

“x” not tested due to lethality of the Bpa substitution.

**Supplementary Table S6.** Cross-linking of Bpa-substituted Rpb4 to Spt5.		
**No.**	**Residue**	**Cross-linked to Spt5**
1	K17	No
2	E19	Yes
3	E21	Yes
4	Q41	No
5	K60	No
6	K75	Yes
**7**	E120	No
8	E124	No
9	N137	Yes
10	K139	Yes

Supplementary Table S7. Cross-linking of Bpa-substituted Rpb7 to Spt5 and Tfa1.

No.	Rpb7 Residue	Cross-linked to Spt5	Cross-linked to Tfa1
1	F17	Yes	No
2	N53	Yes	No
3	Q57	No	No
4	L62	No	No
5	H97	Yes	No
6	E100	Yes	No
7	R142	No	No
8	E148	Yes	No
9	I151	Yes	Yes
10	H158	Yes	No
11	I160	Yes	Yes

Supplementary Table S8. Cross-linking of Bpa-substituted Spt5 to Rpb1, Rpb2, Rpb4 and Rpb7.

No.	Residue	Region	Cross-linked to			
			Rpb1	Rpb2	Rpb4	Rpb7
1	Q16	acidic	No	No	No	No
2	E53	acidic	No	No	No	No
3	E251	linker	No	No	No	No
4	R291	NGN	No	No	No	No
5	R293	NGN	No	No	No	No
6	K296	NGN	No	Yes	No	No
7	R313	NGN	No	Yes	No	No
8	K317	NGN	No	No	No	No
9	K318	NGN	No	No	No	No
10	E338	NGN	No	No	No	No
11	N350	NGN	No	Yes	No	No
12	D354	NGN	No	Yes	No	No
13	E367	NGN	Yes	No	No	No
14	E383	KOW1	No	No	No	No
15	Q433	KOW1	No	No	No	No
16	R458	linker (KOW1-2)	No	No	No	No
17	Q522	linker (KOW1-2)	No	No	No	No
18	R539	KOW2	No	No	No	No
19	D560	KOW2	No	No	No	No
20	E585	KOW3	No	No	No	No
21	E608	KOW3	Yes	No	No	No
22	E642	linker (KOW3-4)	No	No	No	No
23	K654	linker (KOW3-4)	No	No	No	No
24	E672	linker (KOW3-4)	No	No	No	No
25	K706	KOW4	Yes	No	No	No
26	E720	KOW4	No	No	Yes	Yes
27	K737	KOW4	No	No	Yes	No
28	K758	linker (KOW4-5)	No	No	Yes	No
29	K765	linker (KOW4-5)	No	No	Yes	No
30	K778	linker (KOW4-5)	No	No	No	No
31	K785	linker (KOW4-5)	No	No	No	No
32	Q792	linker (KOW4-5)	No	No	No	No
33	D821	KOW5	Yes	No	No	No
35	K834	KOW5	No	No	No	No
36	H843	KOW5	No	No	No	No
37	E854	Linker	No	No	No	No
38	Y1011	CTR	No	No	No	No

**Supplementary Table S9.** Cross-linking of Bpa-substituted Rpb7 to CTR Deleted Spt5.		
**No.**	**Residue**	**Cross-linked to CTR deleted Spt5**
1	I160	x
2	H158	Yes
3	I151	Yes
4	E148	Yes
5	E100	Yes
6	H97	Yes
7	F17	Yes
8	N53	Yes

"x" not tested due to lethality of the Bpa substitution combined with the Spt5 CTR deletion.

SUPPLEMENTARY FIGURE LEGENDS

Supplementary Figure S1. Domains of NusG in bacteria and Spt5 in archaea and eukaryotes. The NGN domain of Spt5 interacts with Spt4 in archaea and eukaryotes.

Supplementary Figure S2. Bpa substitution of a residue of protein of interest and photo-cross-linking in living yeast cells. (A) Plasmid pLH157, which contains genetically engineered *E. coli* tRNA synthetase (Ec-TyrRS) and tRNA (Ec-tRNA$_{CUA}$) genes for incorporating Bpa through nonsense suppression of the TAG codon. (B) Plasmid pGOI-TAG, which bears a gene of interest (GOI) with a TAG codon substituting a normal amino acid codon. This plasmid was transformed through plasmid shuffling into a yeast strain whose genomic GOI is deleted and contains pLH157. (C) Structure of Bpa. (D) Rpb4/7 subcomplex of RNAP II showing the location of Rpb7 F42 (based on PDB 1Y1W). (E) Western blots showing substitution of Rpb7 F42 with Bpa caused cross-linking to Rpb4. Red asterisks indicate bands of cross-linked 3×Flag tagged Rpb4 and 3×Myc tagged Rpb7 (Rpb4+Rpb7), which can be detected with anti-Flag and anti-Myc antibodies.

Supplementary Figure S3. Cross-linking of Bpa-substituted Rpb1, Rpb2, Rpb4 and Rpb7 to interacting proteins. (A-D) Western blots. Sites of Bpa substitutions are shown above the lanes of each blot. Shifted bands presumably caused by cross-linking to Spt5 are marked with red arrow heads. Rpb1 was detected with antibody 8WG16. 3×Myc tagged Rpb2, Rpb4 and Rpb7 were detected with an anti-Myc antibody.

Supplementary Figure S4. Locations of Bpa-substituted residues on a model structure of Spt5. The domains of Spt5 are shown in different colors as indicated. Residues that cross-linked to RNAP II subunits are shown in violet and those that did not cross-link to the subunits are shown in black. See Supplementary Table S8 for a list of all Bpa-substituted residues in Spt5. The model structure of Spt5 was generated by using the I-TASSER server (http://zhanglab.ccmb.med.umich.edu/I-TASSER). Note that the model structure of Spt5 may be disparate from real situation, because even the NGN domain in the model structure is very different from that in the real crystal structure of Spt5 NGN bound to Spt4.

Supplementary Figure S5. Cross-linking of Bpa-substituted Spt5 to Rpb1, Rpb2, Rpb4 and Rpb7. (A-D) Western blots. Sites of Bpa substitutions are shown above the lanes of each blot. Shifted bands presumably caused by cross-linking of Spt5 to the different RNAP II subunits are marked with red arrow heads. 3×Flag tagged Spt5 was detected with an anti-Flag antibody.

Supplementary Figure S1

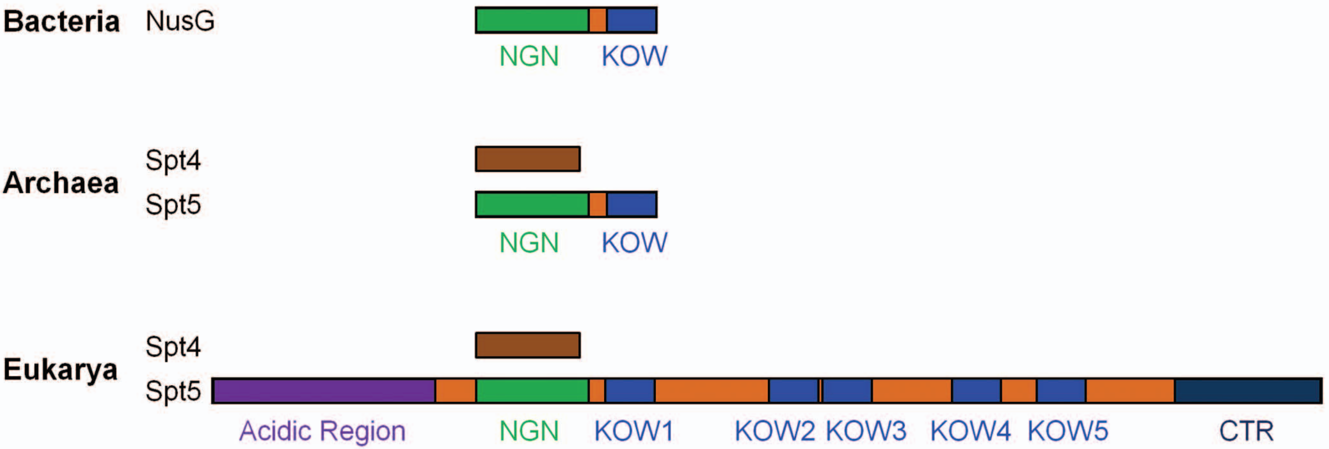

Supplementary Figure S2

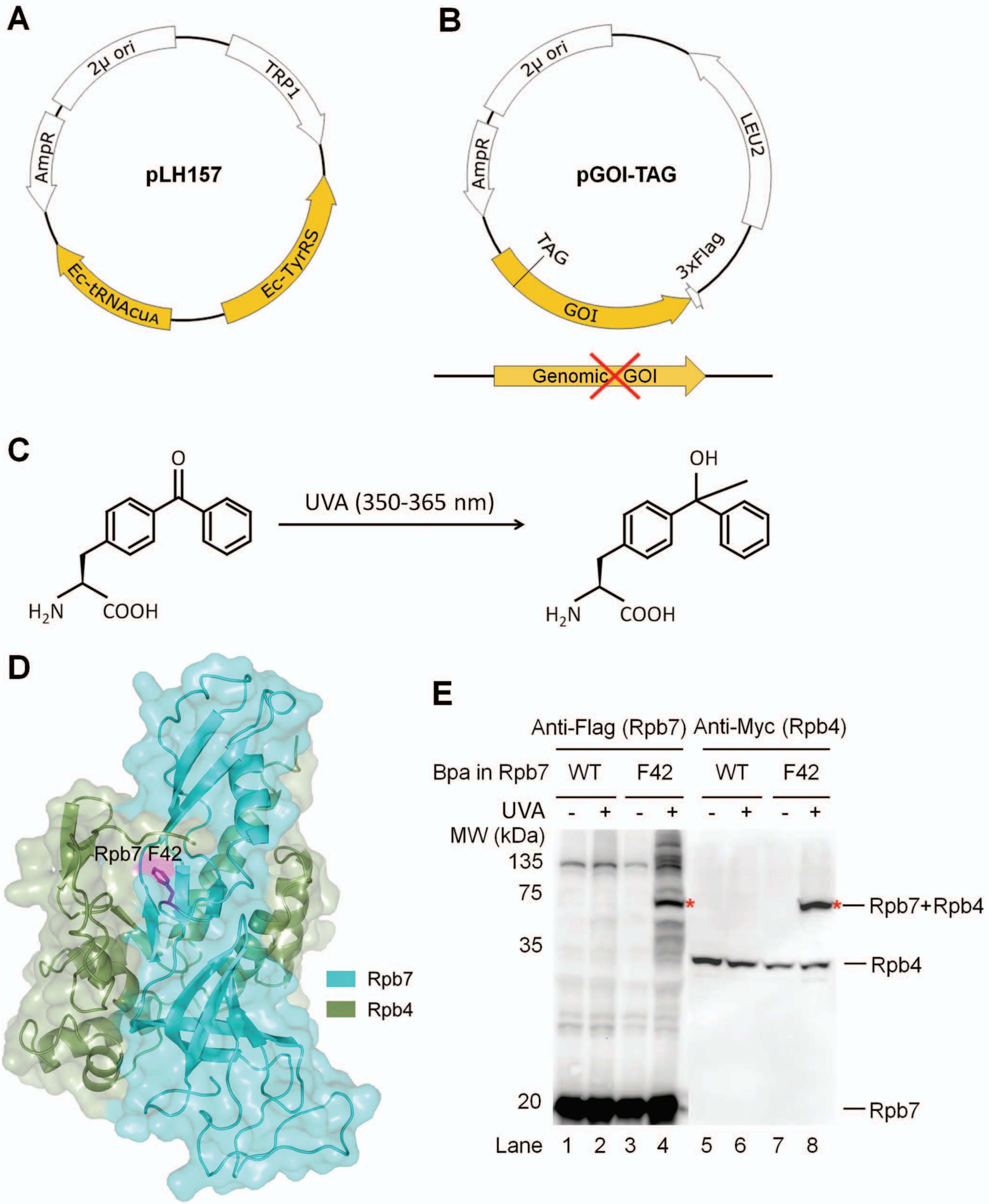

Supplementary Figure S3

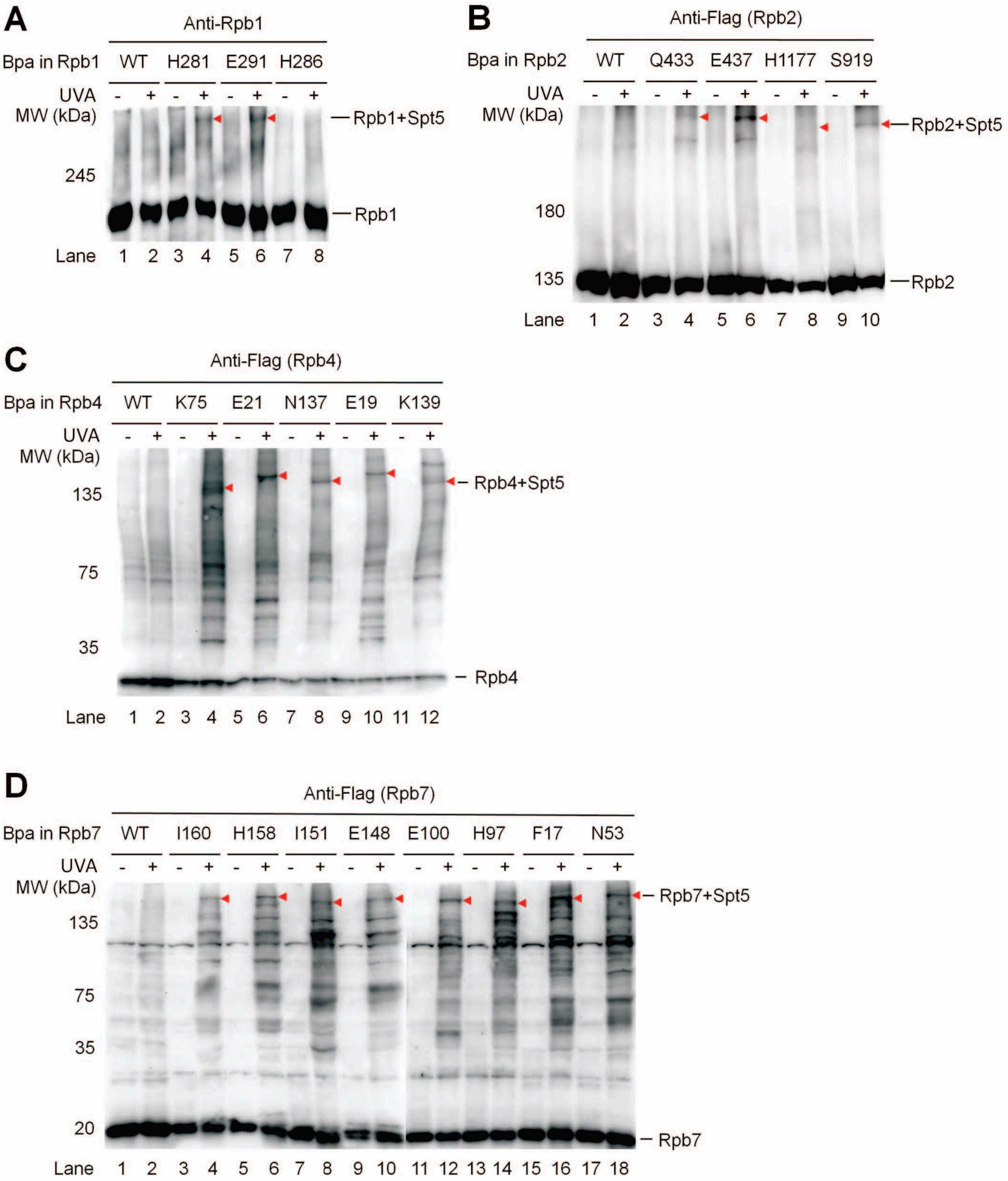

Supplementary Figure S4

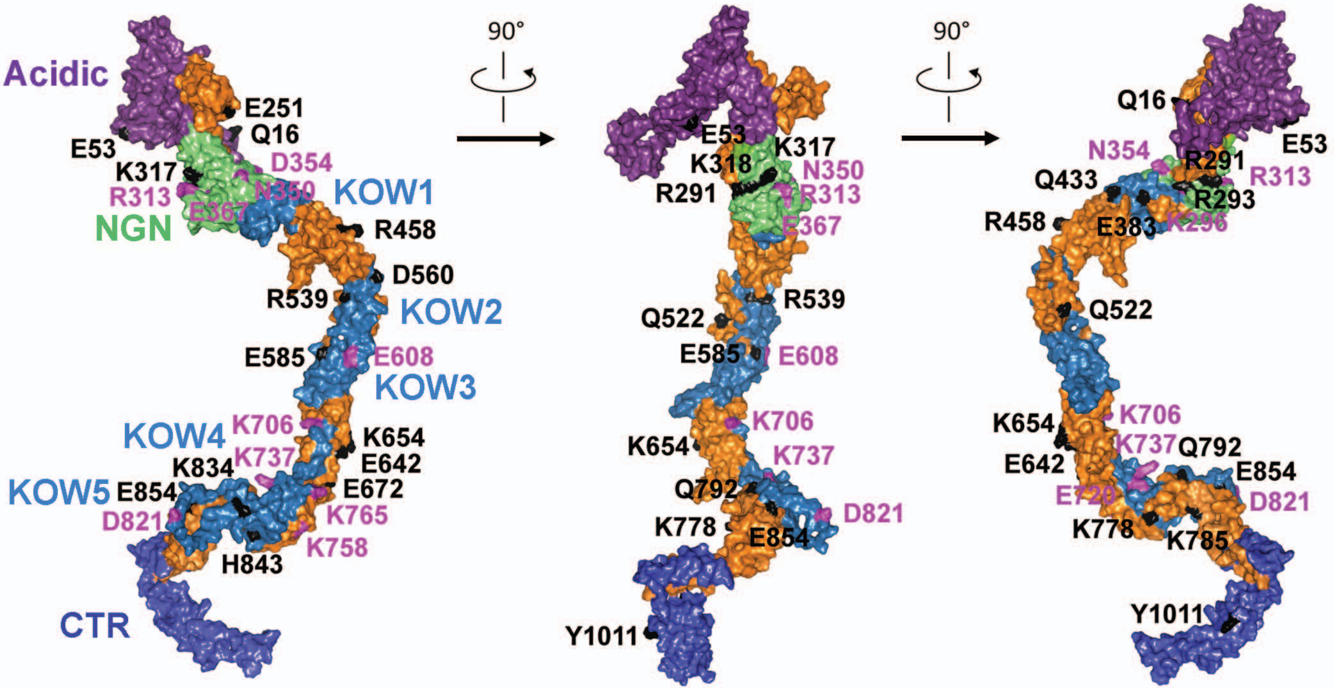

Supplementary Figure S5

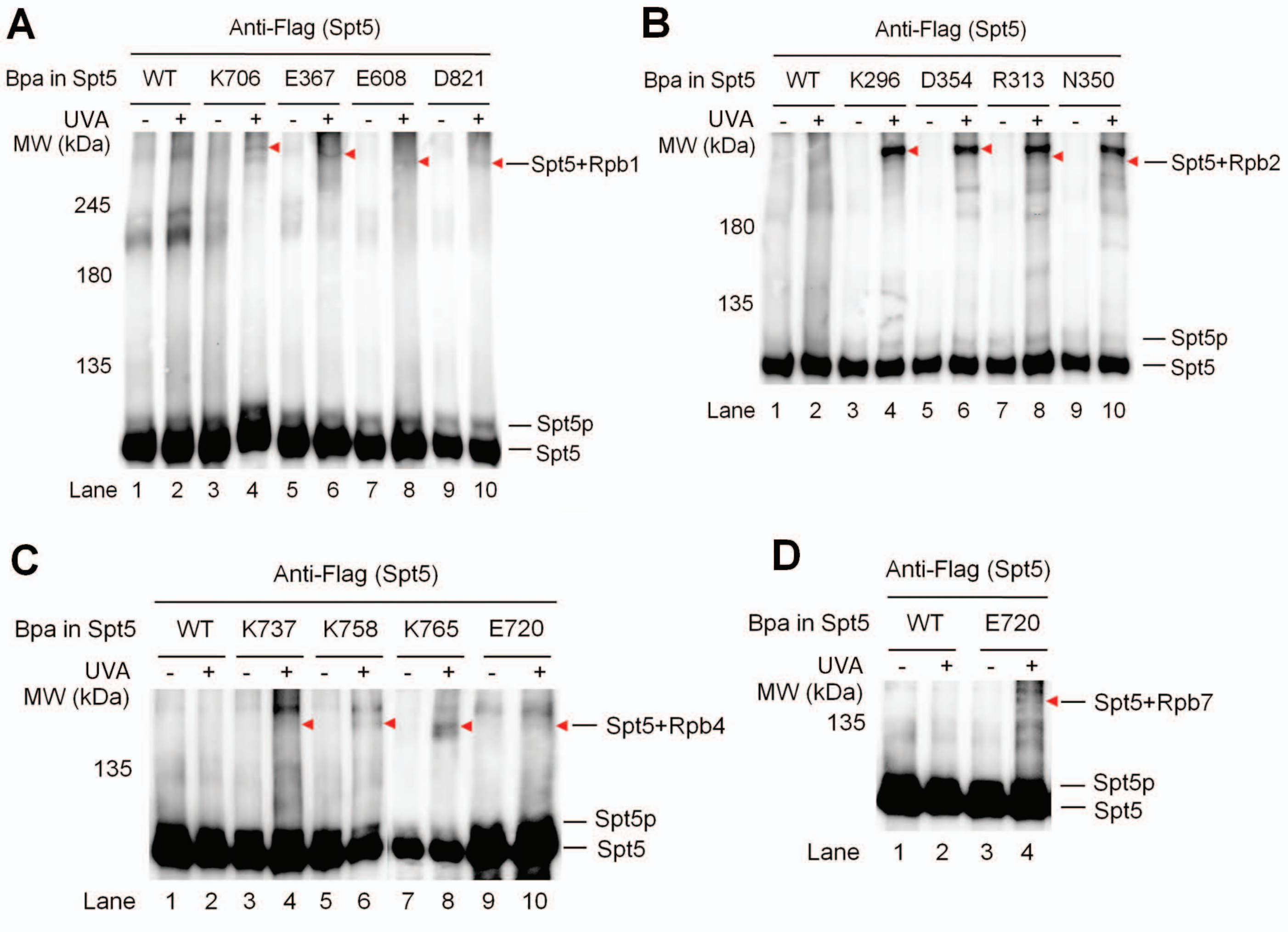

Supplement: SUPPLEMENTARY DATA [file supp_gku333_nar-03675-z-2013-File010.pdf]
